# Investigation of V/III ratio dependencies for optimizing AlN growth during reduced parasitic reaction in metalorganic vapor phase epitaxy

**DOI:** 10.1038/s41598-023-30489-z

**Published:** 2023-02-27

**Authors:** Atsushi Tomita, Takumi Miyagawa, Hideki Hirayama, Yuusuke Takashima, Yoshiki Naoi, Kentaro Nagamatsu

**Affiliations:** 1 Graduate School of Advanced Technology and Science, Tokushima University, 2-1 Minami-Josanjima, Tokushima, Tokushima 770-8506 Japan; 2 Institute of Post-LED Photonics, Tokushima University, 2-1 Minami-Josanjima, Tokushima, Tokushima 770-8506 Japan; 3 RIKEN, Institute of Physical and Chemical Research, Wako, 351-0198 Japan

**Keywords:** Engineering, Electrical and electronic engineering

## Abstract

AlGaN-based ultraviolet (UV) light-emitting diodes (LEDs) are expected to have various applications, including sensing and printing, and light with ultraviolet-C (UVC) wavelengths has a virus inactivation effect. The metalorganic vapor phase epitaxy (MOVPE) method has been used to fabricate LED devices with film control and impurity doping. However, to achieve high luminous efficiency, highly crystalline aluminum nitride (AlN) must be grown in the underlying layer. Although high temperatures are required to grow high-quality AlN for strong migration at the surface, there is a trade-off in the high temperature promoting parasitic reactions. These parasitic reactions are more dominant at a high V/III ratio with more raw material in the case of using the conventional MOVPE. Here, we used jet stream gas flow MOVPE to investigate the effect of V/III ratio dependencies in optimizing AlN growth and without affecting parasitic reaction conditions. As a result, trends of typical AlN crystal growth at V/III-ratio dependencies were obtained. AlN is more stable at a higher V/III ratio of 1000, exhibiting a double atomic step surface, and the crystal orientation is further improved at 1700 °C compared to that at a lower V/III ratio.

## Introduction

AlGaN-based ultraviolet (UV) light-emitting diodes (LEDs) have promising applications in sterilization, sensing, and printing. For example, light with a wavelength of less than 280 nm confirmed the inactivation effect for SARS-CoV-2^1–4^. Metalorganic vapor phase epitaxy (MOVPE) is required to realize AlGaN-based UV-LEDs for several reasons, including thin layer control, impurity doping in n- and p-type semiconductors, and commercial viability. However, dislocations in the AlGaN grown using MOVPE, which are continued from the underlying aluminum nitride (AlN) layer decrease the LED efficiency^5–7^. Additionally, MOVPE AlN growth has two problems that restrict growth conditions required for obtaining ideal growth. The first problem is the growth temperature. AlN growth necessitates a higher Al-adatom migration length in the vicinity of the solid phase on the surface. The AlN growth temperature is much higher than that of GaN, which is optimized at approximately 1100 ℃^8–10^. However, the typical AlN growth temperature achieved by conventional MOVPE is less than 1400 ℃ because high temperatures above 1500 ℃ are rarely achieved in terms of equipment materials and process control. Recently, an MOVPE system with a high temperature for AlN has been developed and high-temperature growth can be realized over 1700 ℃^11,12^.

The second problem is the difference in V/III ratio between the supply and effective growth contribution. Nitride semiconductor crystal growth is generally achieved using MOVPE with a simultaneous supply of group III and V raw materials^13,14^. Therefore, parasitic reactions can occur during raw material transportation^13,14^. These reactions are particularly significant during AlN growth at high temperatures; adduct formation does not contribute to growth in the case of polymerization^15–18^. This adduct formation results in a decrease in growth rate and deterioration in crystalline orientation^13,19^. These adducts are formed as the growth rate slows and crystalline orientation deteriorates owing to the generated dislocations^15,16^. Several researchers have avoided these phenomena by reducing the ammonia gas flow^13,20,21^; however, AlN growth conditions are easily influenced by parasitic reactions. As previously mentioned, Our MOVPE grow AlN at high temperatures without any influence of parasitic reactions^11,12^. Therefore, we have investigated the effect of V/III ratio dependencies for optimizing AlN growth without the effects of parasitic reactions by using jet stream gas flow MOVPE.

## Results and discussion

### The common growth layer in the first AlN layer

**Figure 1:**
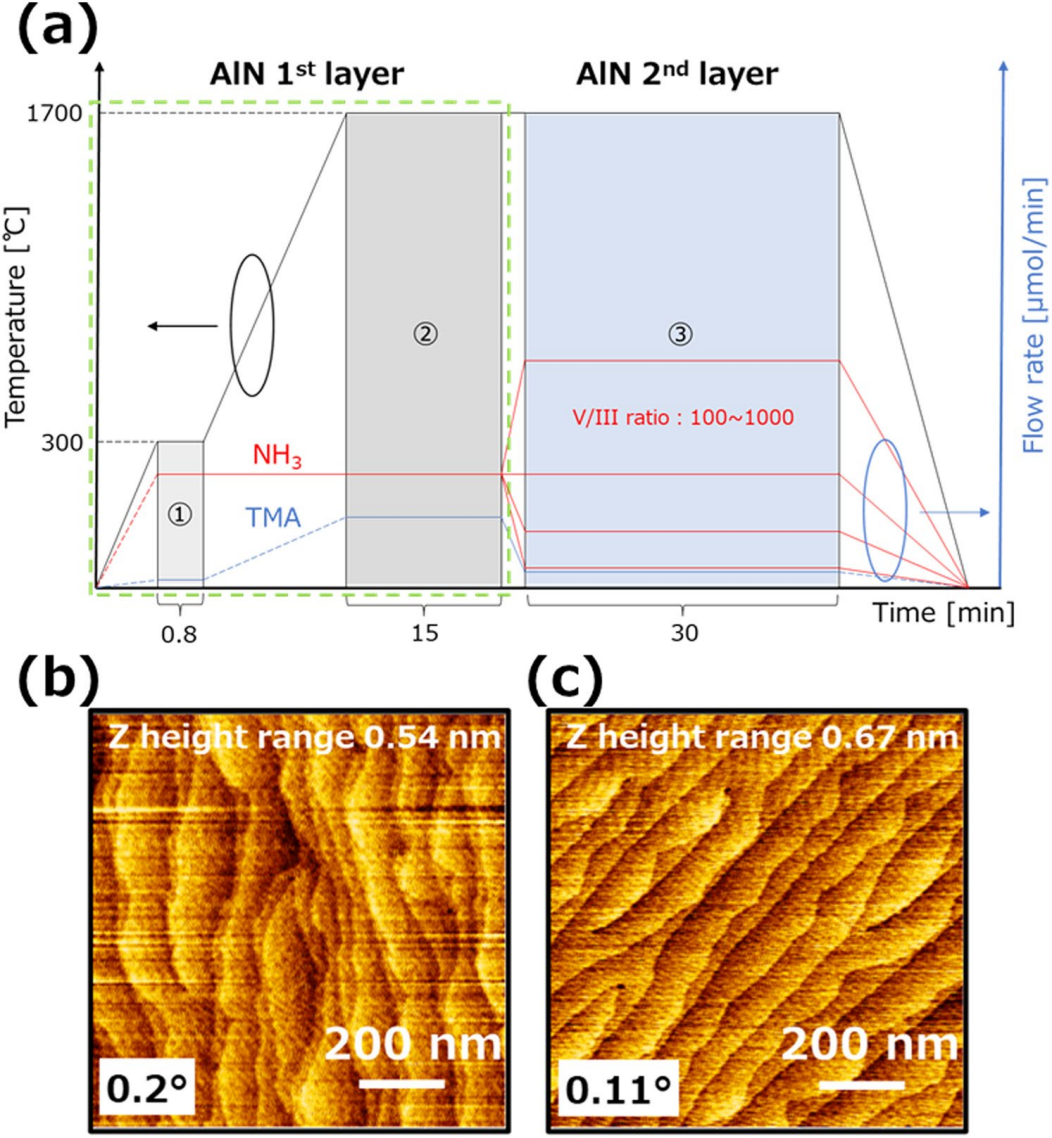
Growth of the first AlN layer. (**a**) The AlN layer’s growth sequence is determined to compare the difference by the V/III ratio. The initial 15 min is a common condition, which is the first AlN layer. (**b**) The AFM image after the first AlN layer grown with sapphire misorientation off-angle of 0.2°. (**c**) The AFM image of the AlN surface after growing the first AlN layer at an off-angle of 0.11°. An atomically flat surface was obtained.

A minimum requirement for comparing AlN growth using several V/III ratios is an atomically smooth surface with atomic steps at the first layer. This is because the transition in the growth mode is primarily affected by this first layer. Figure 1 shows the growth sequence for the AlN layer with a comparison of the V/III ratio used in this study. This growth method involves two steps: the first for common growth and the second for comparison. Therefore, after the common growth mode, a stable surface is required to compare the growth condition of the second AlN layer with ammonia flow rate dependence. The common layer consists of the buffer and the first AlN layers. The growth temperature and trimethyl aluminum (TMA) flow rate were 1700 ℃ and 341 μmol/min, respectively. The growth rate was approximately 12 μm/h and the first-layer thickness was approximately 3 μm after 15 min of growth. Figure 1b shows the atomic force microscopy (AFM) image after the first AlN layer growth on a typical sapphire substrate for AlN growth with an off-angle of 0.2°. An atomically flat surface was obtained. Additionally, the AlN was grown on a low miscut angle sapphire substrate (value of 0.11°) because the migration length increases at high temperature. The surface morphology is smooth (Fig. 1c). These results show that the required AlN condition in the first AlN layer is met for comparison of AlN-layer growth based on V/III ratio difference.

### The confirmation of the influence in parasitic reaction at V/III dependence at 1700 ℃

**Figure 2:**
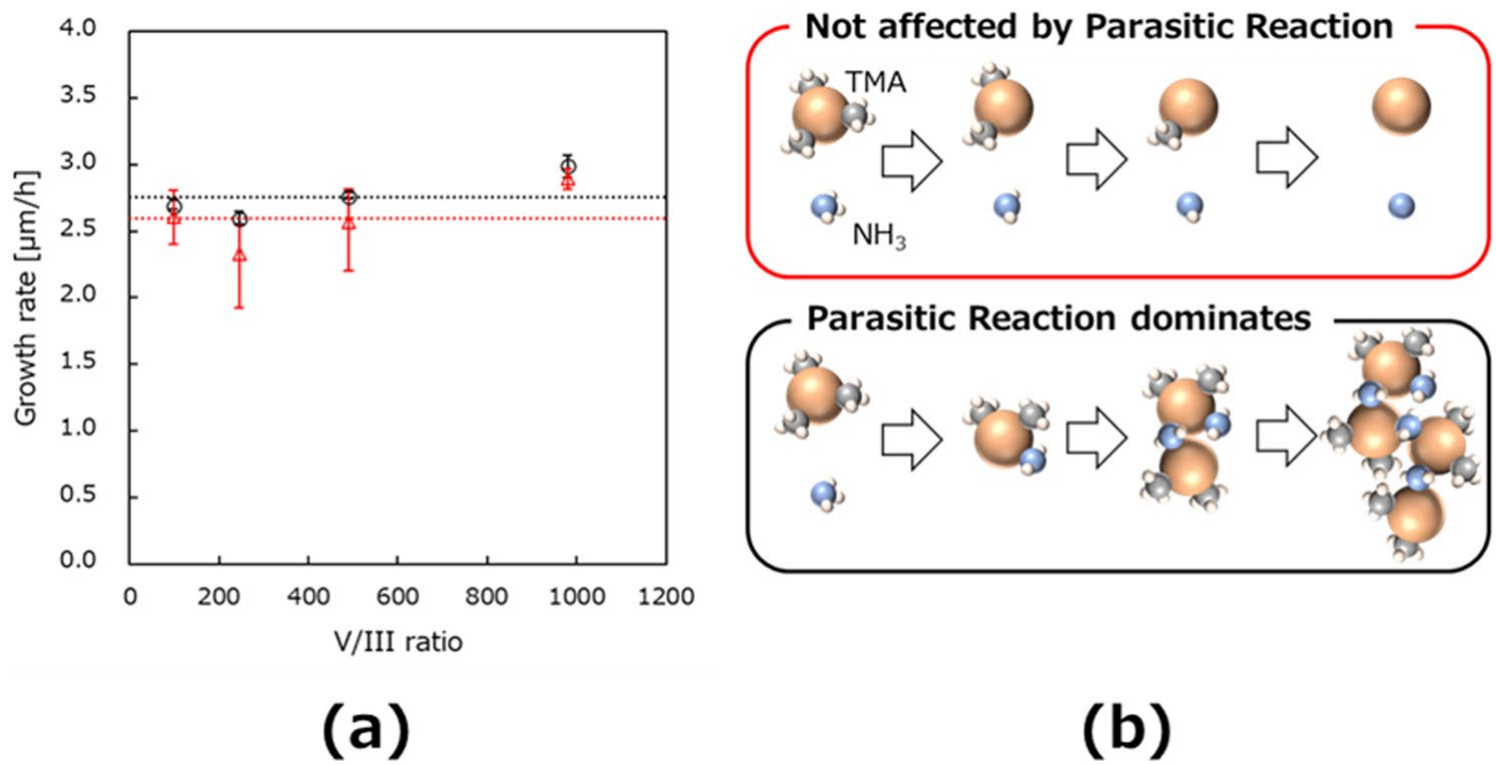
Confirmation of reduction in parasitic reactions during growth. (**a**) The growth rate of the second AlN layer with various V/III ratios (98, 245, 490 and 980). The dotted line represents a guideline for assuming the same growth rate. (**b**) The mechanism of decreasing AlN growth rate^11,15,16^.

The parasitic reaction in AlN growth could be due to the difference in growth conditions and substrates used. Thus, if some growth changes occur, it is necessary to investigate the confirmation because of the reduced parasitic reactions using jet stream gas flow MOVPE. Figure 2 shows the AlN growth rate at several ammonia flow rates at sapphire misorientation angles of 0.2° and 0.11° at 1700 ℃. These setting values in V/III ratios of 98, 245, 490, and 980 with the changing ammonia flow rate are aimed at 100, 250, 500, and 1000 values, respectively. Both growth rates at 0.2° and 0.11° are stable until the V/III ratio of 500. Additionally, the V/III ratio of 1000 is slightly higher than that of other V/III ratios. The TMA flow rate is constant at the value of 91 μmol/min in the second AlN layer at each V/III ratio.

Moreover, the difference in growth rate by misorientation angle is observed at 0.2° and 0.11°. If the adduct formation is the main reaction during transportation, the growth rate decreases with an increasing ammonia flow rate, as shown in the downside of Fig. 2b. The AlN was grown, as shown at the upper side of Fig. 2b from the stable growth rate with the ammonia flow dependency shown in Fig. 2a. This is due to the high gas velocity of transport while maintaining space between Al and N sources^11^. Thus, the influence of parasitic reactions is confirmed enough to be reduced in this study.

### The investigation of the V/III ratio dependence of the second AlN layer

**Figure 3:**
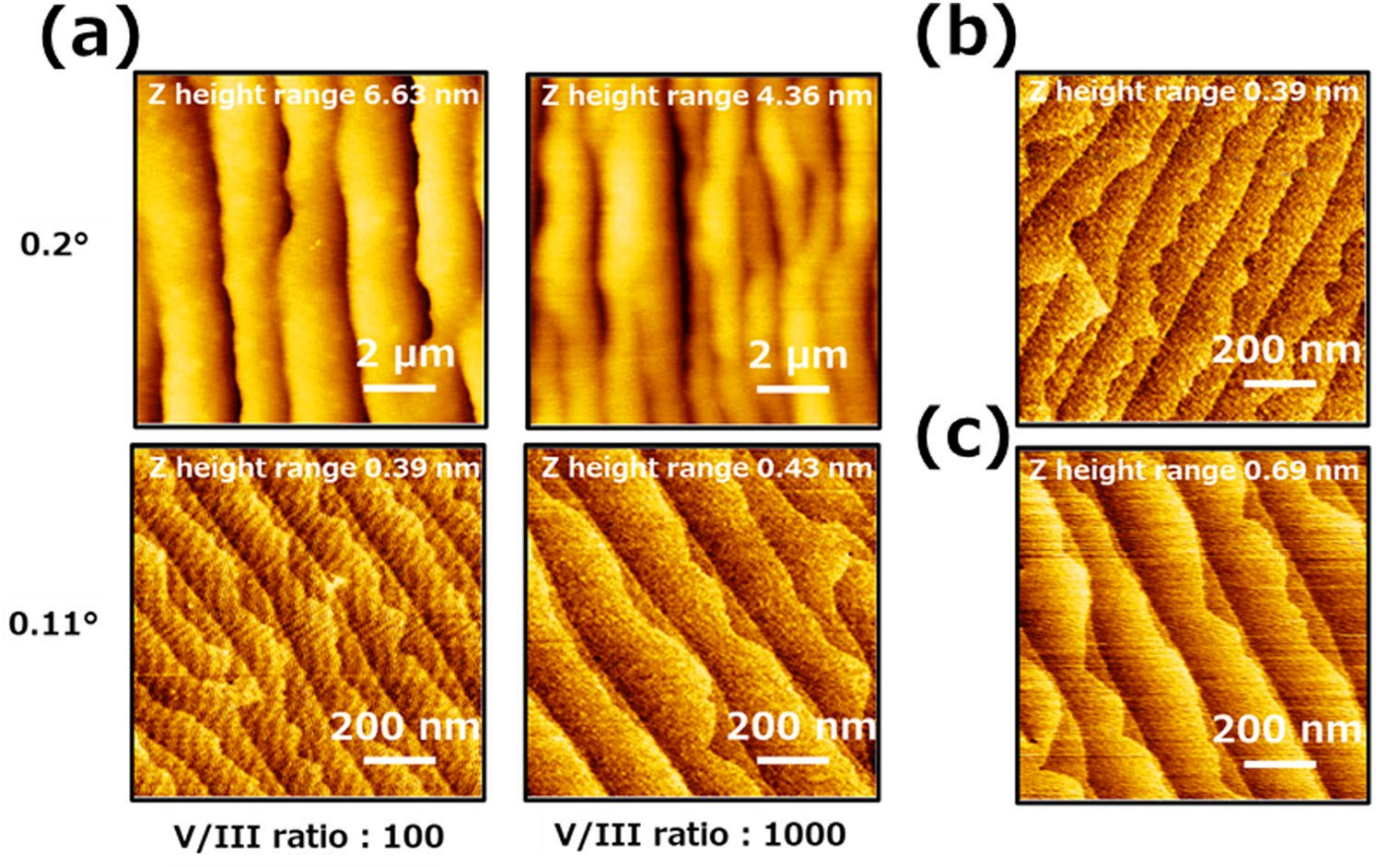
The AFM images with the V/III ratios of 100 and 1000 on a sapphire off-angle of 0.2° and 0.11°. (**a**) The second AlN layer growth for 30 min. (**b**) The second AlN layer growth for 0 min on a sapphire off-angle of 0.11°. (**c**) The second AlN layer growth for 0.2 min at V/III ratio 1000 on a sapphire off-angle of 0.11°.

First, a detailed analysis of V/III ratio dependencies with the grown AlN at 1700 ℃ was performed based on the confirmation of reducing parasitic reaction at 1700 ℃, and the required atomically flat AlN surface for stable growth mode was obtained. Figure 3 shows the AFM images after the second AlN layer with V/III ratios of 100 and 1000, as shown in the growth sequence in Fig. 1a. Step bunching was observed when a sapphire substrate with a miscut angle of 0.2° was used. Additionally, the step-bunching interval was clarified, narrowing with increasing V/III ratio. However, atomic steps were observed when a sapphire substrate miscut angle of 0.11° was used, without V/III ratio dependence. The atomically step interval became significant with an increasing V/III ratio. Furthermore, the atomic steps of the V/III ratio of 100 and 1000 were found in the monolayer and bilayer from the step interval. According to a previous report, three-dimensional growth is dominant when the V/III ratio is high. This trend is similar to the short migration length effect^13^. Different surface atom structures change the formation energy. Therefore, the growth mode when step bunching occurs is expected to be different from that of atomic steps. Moreover, the growth mode can adapt step-flow despite forming step-bunching based on the growth rate results in shown Fig. 2a.

Meanwhile, the atomic steps at 0.11° is a contrary result in those results. This result reduced the desorption of the adatom from the surface in case of a high V/III ratio. This phenomenon of AlN in the atomic step is of two types; there is a difference in step edge energies at each step^22,23^. Additionally, bilayer atomic steps are expected to be better for crystalline orientation because atomic steps are going to be unified with high linearity steps.

Second, we investigated the changes from monolayer to bilayer on the surface at different V/III ratios. The growth time of the second AlN layer was changed to 0 and 0.2 min, following the growth sequence shown in Fig. 1a. Figure 3b shows an AFM image without the second layer growth that maintains a monolayer and atomically smooth surface (Fig. 1c). The AFM image of the second layer in Fig. 3c at a growth time of 0.2 min indicates that a double step has already formed. The results show that the surface morphology transited to double step at the initial growth of only approximately 36 atomic layers.

**Figure 4:**
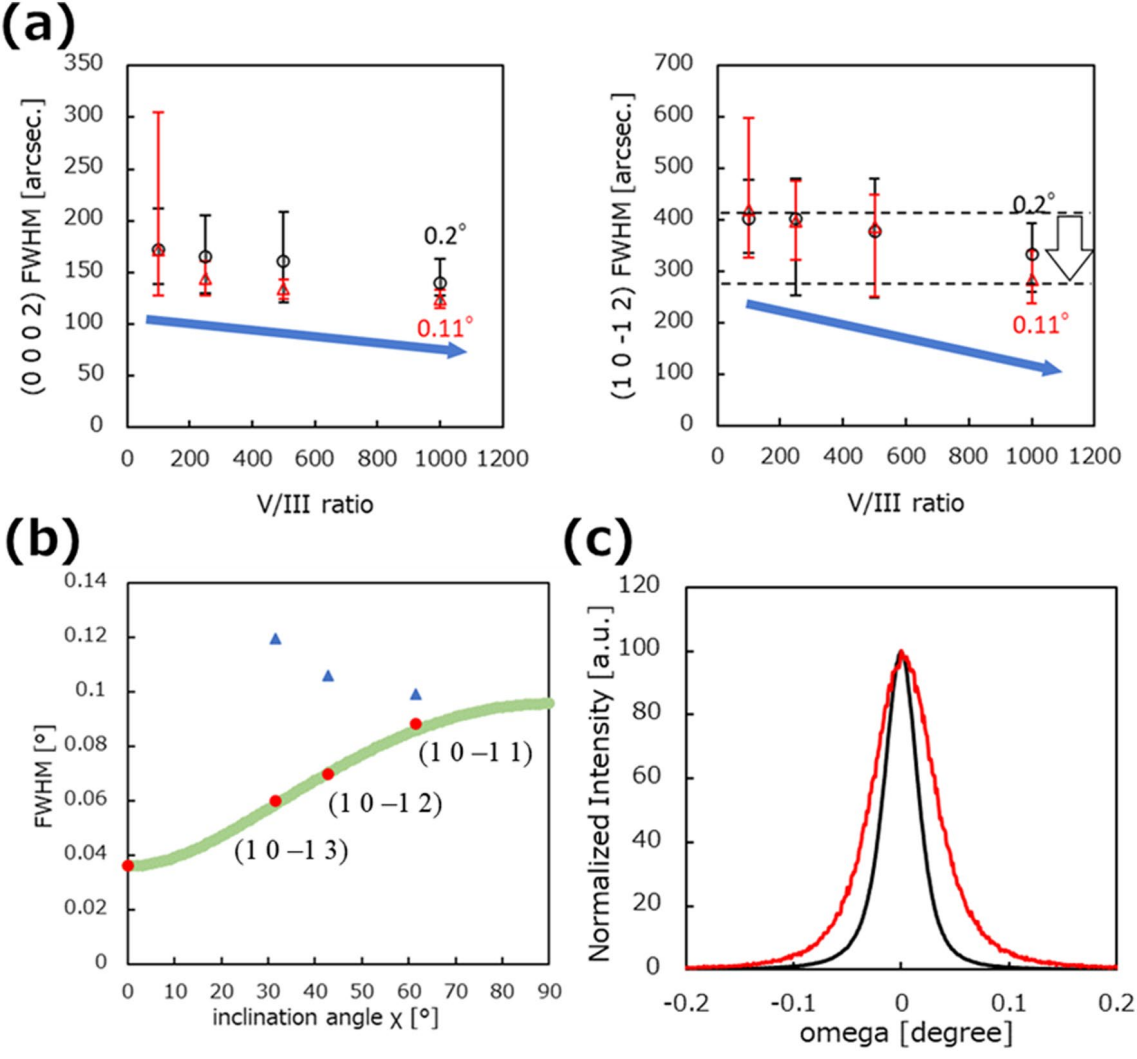
FWHM of XRC measurements obtained for crystalline orientation after second AlN layer growth. (**a**) FWHM of (0002) (left) and (10–12) (right) as a function of V/III ratio dependency for AlN grown on 0.2° and 0.11° miscut-angle sapphire substrate. (**b**) FWHM of XRC φ (blue triangle) and ω (red circle) scans at various inclination angles (χs). The green theoretical curve was fitted using Eq. (1) according to Refs. 24 and 25 using the FWHM of the ω scan. (**c**) XRC FWHM spectrum 20 mm from the center of a layer grown on sapphire with a miscut angle of 0.11° and V/III ratio of 1000. The black line indicates the (0002) plane, and the red line indicates the (10–12) plane.

X-ray rocking curve (XRC) measurements were performed for each sample at five in-plane points to determine the crystalline orientation. The full width at half maximum (FWHM) values of (0002) and (10–12) improved with increasing V/III ratio (Fig. 4a). The average FWHM values of (0002) and (10–12) with a V/III ratio of 980 on a 0.11° sapphire substrate were 124 and 284 arcsec, respectively. The crystalline orientation of AlN grown on a 0.11°-miscut substrate is slightly better than that with a miscut angle of 0.2°. These results show that a high V/III ratio is required for AlN growth at high temperatures to optimize growth conditions. The V/III ratio-dependent results in this study show a decreasing trend, contrary to a previous study in which the FWHM increased significantly with increasing V/III ratio^13^. To confirm this decreasing trend, we performed ω and φ scans with increasing χ at a point 20 mm from the center with an off-angle of 0.11° and a V/III ratio of 1000. Figure 4b shows the results along with the theoretical curve of FWHM obtained from Eq. (1), which is derived by Lee et al.^24^ and Pantha et al.^25^ With increasing χ, the values of each FWHM approach each other, and at 90°, they are equal. Figure 4b shows that the FWHM of the ω scan increases and the FWHM of the φ scan decreases with increasing χ. The FWHM of the ω scan is located on the theoretical curve in the χ direction, and the results show the value of twist along the (1–100) plane. Therefore, the FWHM results for the (10–12) plane indicate the tendency of a twist along the (1–100) plane correctly. As a result, the crystallinity measured using XRD was confirmed to improve with increasing V/III ratio. Figure 4c shows the FWHM spectrum of the ω scan obtained at a V/III ratio of 1000 using a sapphire miscut angle of 0.11°. In Fig. 4c, the FWHM values of (0002) and (10–12) are 134 and 234 arcsec, respectively.

**Figure 5:**
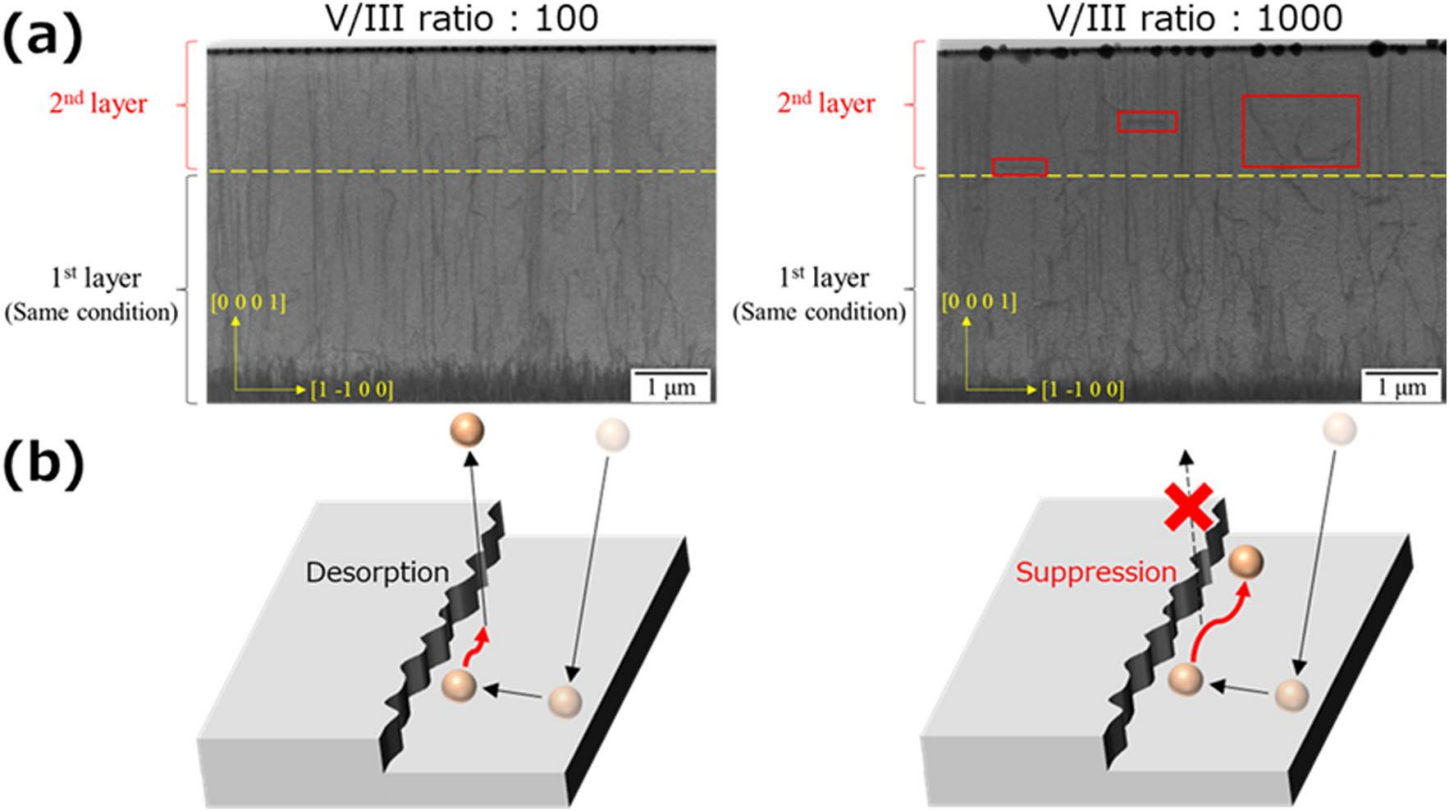
The difference in the behavior of threading dislocations by V/III ratio. (**a**) The measurement of the dislocation densities by field-emission TEM images. The TEM images at both V/III ratios were measured in the same regions. The measurement volume is 6.9 μm × 260 nm × 4.8 μm. The yellow dotted line represents the boundary between the first and second layers. (**b**) Growth models at different V/III ratios.

The behavior of dislocations at V/III ratios of 100 and 1000 was examined using TEM measurements. Figure 5a shows field-emission TEM images. The sample was processed to a depth of approximately 260 nm for measurement. At a V/III ratio of 100, the threading dislocations propagate approximately straight along the c-axis direction. However, in the case of a V/III ratio of 1000, some threading dislocations (indicated by red lines in Fig. 5) have propagated toward the m-axis in the AlN second layer. Additionally, total threading dislocation densities are estimated as $1.8\times {10}^{9}$ and $8.8\times {10}^{8} {\mathrm{cm}}^{-2}$ using cross-sectional TEM images. The difference in the behavior of the threading dislocations could be due to the growth models in Fig. 5b. High-temperature growth at 1700 °C is the region wherein the growth rate decreases^12^. This could be adatom desorption during migration due to the high thermal kinetic energy. For low V/III ratios with low NH_3_ supply, adatoms desert after a short migration. However, at high V/III ratios, the high nitrogen partial pressure due to the large amount of NH_3_ suppresses adatom desorption, allowing further surface migration. Adatoms with suppressed desorption migrate one-dimensionally along the kink after reaching the step edge, which is ideal for crystal growth. Figure 2a shows that the growth rate with a V/III ratio of 1000 is slightly higher than that with a V/III ratio of 100. Therefore, growth along the kink (i.e. along the m-axis) is promoted, and dislocations are considered to have propagated along the m-axis direction.

## Conclusion

We investigated the dependence of AlN growth on V/III ratio without the influence of parasitic reactions using jet stream gas flow MOVPE. The XRD-FWHM value decreases with increasing V/III ratio in this study, which is significantly different than previous reports wherein parasitic reaction influences were involved^13,14^. These data show that the parasitic reactions in previous studies limit the ability to obtain optimized AlN growth conditions. Additionally, after solving the parasitic reaction problem, the obtained XRC FWHMs for (0002) and (10–12) were 124 and 284 arcsec, respectively. Moreover, the double atomic step is stable in the AlN growth.

## Methods

All AlN samples were grown using MOVPE on sapphire substrates. Sapphire substrate with miscuts of 0.11° and 0.2° toward the m-axis were used. The pressures of Al and N are TMA and ammonia, respectively, and the carrier gas is hydrogen. The AlN buffer layer growth temperature and growth time are 300 ℃ and 48 s, respectively. The total gas flow rate of the buffer and second AlN layer were 18 and 26 slm, respectively. The thickness of the grown AlN was measured using an optical interference film thickness meter. The atomic layer and surface roughness in AlN was measured using AFM (HITACHI Corporation 5500 M). The XRC measurement was obtained using a Smart Lab (Rigaku Corporation). Additionally, the obtained FWHM data confirm the fitting as follows^24,25^.

1 $$\beta =\sqrt{{\left({\beta }_{\mathrm{tilt}}\mathrm{cos}\chi \right)}^{2}+{\left({\beta }_{\mathrm{twist}}\mathrm{sin}\chi \right)}^{2}}$$

where $\chi$ is the angle between the reciprocal lattice plane (hkl) and (0001) plane, and ${\beta }_{\mathrm{tilt}}$ and ${\beta }_{\mathrm{twist}}$ are the FWHM of tilt and twist, respectively, measured using the XRC measurements.

## Data Availability

The data that support the findings of this study are available from the corresponding author upon reasonable request.
